# Balloon dilation improves both the hearing level and the quality of life in patients suffering from obstructive Eustachian tube dysfunction

**DOI:** 10.1007/s00405-023-08231-2

**Published:** 2023-09-19

**Authors:** Leena Pöyhönen, Juha Silvola, Dennis Poe, Markus Rautiainen, Ilkka Kivekäs

**Affiliations:** 1 Department of Ear and Oral Diseases, Tampere University Hospital, P.O. Box 2000, 33521 Tampere, Finland; 2 Department of Otorhinolaryngology, Faculty of Medicine, Akershus University Hospital, Akershus and University of Oslo, Campus AHUS, Oslo, Norway; 3 Department of Otolaryngology, Boston Children’s Hospital, Boston, MA USA; 4 Faculty of Medicine and Life Sciences, Tampere University, Tampere, Finland

**Keywords:** Eustachian tube, Balloon dilation, Quality of life, Hearing loss, Tuboplasty

## Abstract

**Purpose:**

Chronic obstructive Eustachian tube dysfunction (OETD) can lead to tympanic membrane (TM) retraction and middle ear effusion (MEE) which can cause conductive hearing impairment, which among other ear symptoms can lower the quality of life (QoL). In this prospective study we assess hearing results and subjective changes in QoL following balloon Eustachian tuboplasty (BET) in treatment of OETD.

**Methods:**

Totally 25 ears with TM retraction and 18 ears with MEE due to chronic OETD underwent BET as the sole intervention. Outcomes including otoscopy, ability to perform the Valsalva maneuver, tympanometry, audiometry, Eustachian tube inflammation scale and the Glasgow Benefit Inventory questionnaire (GBI) were obtained on all patients preoperatively and 6 months postoperatively.

**Results:**

Hearing thresholds improved statistically significantly (*p* < 0.05) with means of 3 dB in the TM retraction group and 9 dB in the MEE group. Total GBI results indicated a positive influence on patients’ QoL. Valsalva success rate was 80% in patients with TM retraction and 67% in patients with MEE. Tympanometry results improved in 50% of TM retraction patients and in 33% of MEE patients.

**Conclusions:**

Here we demonstrated that the BET has a positive impact on patients’ conductive hearing loss and QoL in patients with TM retraction or MEE. Results were better in TM retraction group than in MEE group.

## Introduction

Obstructive Eustachian tube dysfunction (OETD) is a medical disorder that affects 4.6% of the adult population [1]. Patients with chronic OETD often experience subjective ear sensations such as pressure or pain in the ear or clogged sensations or hearing loss. Chronic OETD can lead to tympanic membrane retraction and middle ear effusion, which can cause conductive hearing impairment that may range from mild (commonly) to maximal (more severe scenario).

Most reports of improvement in hearing from balloon Eustachian tuboplasty (BET) have used the patients’ own assessment of their symptoms. Pre- and postoperative pure tone audiometry has been documented only in a minority. Gürtler et al. [2] reported a cohort of 21 patients in which air-bone gaps showed a statistically positive outcome after BET. McMurran et al. [3] also showed improvement post-operatively in 75% of 20 patients with OETD.

Evaluation of the effectiveness of BET has principally focused on clinical outcomes that provide information about OETD, but are not able to evaluate its significance to a patient’s quality of life. Patients’ subjective improvement can be assessed by relief of severity or frequency of symptoms and also with a validated disease-specific questionnaire 7-item Eustachian Tube Dysfunction Questionnaire (ETDQ-7) [4]. Ear symptoms due to chronic OETD may impair quality of life and its impact on quality of life has been evaluated to be comparable to moderate asthma or gastroesophageal reflux disease [5]. Measuring quality of life ensures that treatment and evaluation focus on the patient rather than the disease. This should be taken into account in evaluation of the patient’s overall benefit from BET.

The changes in quality of life after treatment can be measured with a validated generic patient-recorded outcome measure, Glasgow Benefit Inventory (GBI) [6], which is widely used in otorhinolaryngological interventions and it makes possible to comparing of impact of different surgical interventions. The study of Bast et al. [7] showed that BET has a significant improvement in the total GBI score and in subscores of general and physical health 6–18 months postoperatively in 30 patients with chronic OETD.

The available evidence indicates that BET can provide long-term benefit in the treatment of chronic OETD. Most of the studies have been retrospective, but there have been a good number of prospective studies. A recent systematic review by Luukkainen et al. [8] showed that BET can improve patients’ subjective symptoms in 73–98%, otoscopic findings in 90% and success in Valsalva maneuver in 80–98% of patients. Tympanometry improved in 24–54% and tubomanometry in 28–43% of patients in 12 month follow-up. Another systematic review by Huisman et al. with shorter follow-up time supported these findings [9]. There have been two randomized-controlled studies comparing BET vs nasal steroid spray, both of which demonstrated efficacy of BET up to mean follow-up of 12 and 29 months [10, 11].

A number of studies of BET included adjunctive procedures (eg. other otologic or nasal operations or medical managements), which introduced a confounding factor in evaluating the effectiveness of BET alone. This prospective study was designed to study the efficacy of BET as the sole intervention in patients with chronic OETD using objective clinical outcomes and subjective quality-of-life questionnaires.

## Materials and methods

### Study design

A prospective study recruiting adult patients with chronic OETD treated with balloon dilation was conducted in the Department of Otorhinolaryngology in Tampere University Hospital (TAUH), Tampere, Finland. The study design was approved by the ethical review board of the Pirkanmaa hospital district (Tampere University Hospital, Tampere, Finland) and informed consent was obtained from all participants.

The inclusion criteria were: (1) age 18–70 years, (2) unilateral or bilateral non-adherent tympanic membrane (TM) retraction or persistent middle ear effusion (MEE) for 3 months or longer and/or (3) recent extrusion of a tympanostomy tube in which there was a history of multiple tympanostomy tubes, but prompt recurrence of otologic effects of OETD upon extrusion and (4) intact tympanic membranes, absence of tympanostomy tubes. Exclusion criteria included: history of long-term sinusitis (persistent for more than 2 months), nasal polyps, cholesteatoma, chronic otitis media, chronically infected or hypertrophied adenoid contacting torus tubarius, severe systemic disease, craniofacial abnormalities, history of traumatic fracture of the skull base, radiation therapy of head and neck.

### Preoperative examinations

During the first visit, candidates’ ears were examined with otomicroscope and subjects were included based on the above criteria. Ears were divided into two groups: Group (1)—tympanic membrane (TM) retraction and Group (2)—middle ear effusion (MEE).

Tympanometry was performed pre- and post-operatively (Titan by Interacoustics, Audmet Oy, Helsinki). Postoperative results were interpreted as success if tympanogram type B or C improved to type A or type B improved to type A or C curve. Retracted TMs were not permanently adherent to promontorium, in some cases there was adhesion to incus. Tympanometry was performed before and after Valsalva maneuver and once again after swallowing.

The ability to perform a Valsalva maneuver was recorded as positive only when it could be verified objectively with otomicroscopic examination. Pure tone air conduction (AC) and bone conduction (BC) audiometry with appropriate masking was performed preoperatively and 6 months postoperatively. Pure tone averages (PTAs) were calculated using 4-tone averages (0.5, 1, 2, and 4 kHz).

Cone beam computed tomography (CBCT) was performed to examine for disease of the paranasal sinuses and to study the anatomy of the Eustachian tube (ET) and temporal bone. The nasopharynx, mucosal inflammation and opening of the ET were evaluated with nasopharyngeal videoendoscopy under topical spray anesthesia with 4% lidocaine and oxymetazoline. The severity of the mucosal inflammation in the ET orifice was scored according to the ET inflammation classification instrument [12] from normal to mild (scores 1–2) and from moderate and severe (scores 3–4) inflammation.

The impact of surgery on quality of life was measured using the Glasgow Benefit Inventory questionnaire (GBI). The GBI scores depict the positive or negative impact of BET on the patient’s QoL compared to the condition before the operation. The test contains 18 questions and the response to each question is placed on a five-point scale ranging from a large deterioration to a large improvement in health status. The GBI consists of a total score and three subscores (general, social support, and physical health). The total score is transposed onto a benefit scale ranging from –100 (maximal negative benefit), through 0 (no benefit), to + 100 (maximal positive benefit).

### Technique of ET balloon dilation

Patients underwent balloon Eustachian tuboplasty under general anesthesia without any other operation. The technique of BET is described precisely in the study by Silvola et al. [13]. A balloon catheter with 6 mm diameter and 16 mm length catheter was used (Acclarent, Inc, Menlo Park, California, USA). It was inserted through a 70 degree guide catheter into the full length of the lumen of the cartilaginous Eustachian tube and inflated two times up to 12 atm for 1 min each time. Patients were recommended to perform the Valsalva maneuver repeatedly several times per day from the first postoperative day at least until the first postoperative control.

### Postoperative follow-up

Postoperative follow-ups were at 1–2 weeks after tuboplasty (clinical examination, tympanometry, Valsalva, GBI questionnaire), after 3 months (clinical examination, tympanometry, Valsalva, GBI-questionnaire) and after 6 months (clinical examination, tympanometry, Valsalva, audiometry, GBI questionnaire). IBM SPSS Statistics 22.0 was used for the statistical analyses (Crosstabs, Fisher, McNemar, Wilcoxon).

## Results

43 ears were included in the study from 33 patients [14 female, 19 male, mean age 40 years (range 22–68)]. Ears were divided into two groups, Group 1 included 25 ears with TM retraction and Group 2 included 18 ears with middle ear effusion. Demographics are presented by ears in Table 1.

**Table 1:** Demographics of patients and preoperative ear symptoms in groups with tympanic membrane (TM) retraction and ears with middle ear effusion (MEE)

	Group 1: TM retraction (N 25)	Group 2: MEE (N 18)
Background information		
Childhood history of recurrent OME	15 (60%)	9 (50%)
Pollen allergy	6 (24%)	6 (33%)
Gastroesophageal reflux disease	1 (4%)	0 (0%)
Smoking	6 (24%)	7 (39%)
History of grommets	21 (84%)	14 (78%)
Preoperative ear symptoms		
Clogged ear	21 (84%)	8 (44%)
Hearing loss	12 (48%)	7 (39%)
Ear pain	5 (20%)	1 (6%)
Recurrent OME	16 (64%)	14 (78%)

Clinically significant improvement was defined differently for the two groups: Group 1—ears that normalized tympanograms from type B or C to A and Group 2—ears that became aerated. Healthy middle at 6 month follow-up was detected in 84% in Group 1 and 56% in Group 2. Preoperatively the objective Valsalva maneuver success rate was 28% in both groups. At 6 months postoperatively, statistically significant improvement was seen with 80% in Group 1 (*p* < 0.001)and 67% in Group 2 (*p* = 0.016). Improvement was already detected at the first postoperative visit, but it showed further improvement by 6 months. The ET inflammation scale improved in both groups postoperatively and the difference was statistically significant in both groups (Table 2).

**Table 2:** Clinical findings at preoperative visit and at postoperative visits (*TM* tympanic membrane, *MEE* middle ear effusion, *ET* Eustachian tube)

	(*N*)	1–2 weeks	3 months	6 months	*p* value**
Group 1: TM retraction 25 ears					
Air in the middle ear	25	22 (88%)	22 (88%)	21 (84%)	
Middle ear effusion	0	3 (12%)	1 (5%)	4 (16%)	
Valsalva positive	7 (28%)	16 (64%)	19 (76%)	20 (80%)	< 0.001
A tympanometry	2 (8%)	10 (40%)	14 (56%)	12 (48%)	
B tympanometry	6 (24%)	6 (24%)	4 (16%)	6 (24%)	
C tympanometry	14 (56%)	5 (20%)	4 (16%)	4 (16%)	
ET inflammation scale 1–2	15 (60%)	–	–	23(92%)	0.021
ET inflammation scale 3–4	10 (40%)	–	–	2 (8%)	
Group 2: MEE 18 ears					
Air in the middle ear	0	6 (33%)	8 (44%)	10 (56%)	
Valsalva positive	5 (28%)	11 (61%)	11 (61%)	12 (67%)	0.016
A tympanometry	1 (6%)*	2 (11%)	5 (28%)	3 (17%)	
B tympanometry	15 (83%)	14 (78%)	7 (39%)	9 (50%)	
C tympanometry	2 (11%)	1 (6%)	6 (33%)	6 (33%)	
ET inflammation scale 1–2	8 (44%)	–	–	15 (83%)	0.016
ET inflammantion scale 3–4	10 (56%)	–	–	3 (17%)	

*Patient with both ears affected did Valsalva maneuver before tympanometry measurement

**p value between preoperative and 6 months postoperative results

Tympanometry outcomes improved in 11/22 (50%) of ears in Group 1 and in 6/18 (33%) of ears in Group 2. Eight ears with type C and three ears with type B tympanogram improved to type A in Group 1. One ear with type A and two ears with type C tympanogram in Group 1 deteriorated after BET and resulted in a type B by the 6 month follow-up. In Group 2, all six ears with improved tympanometry had type B tympanograms preoperatively. Postoperatively three of them had type A and three of them had type C tympanogram. One ear with preoperative type A tympanogram changed to type C tympanogram (Table 2).

In Group 1, there were two ears with A tympanometry preoperatively. One of these patients got worse during 6 month follow-up, tympanometry changed to type C in first postoperative control and type B in 3 and 6 months, but still with no effusion in tympanic cavity. The other Group 1 patient with type A preoperatively did not undergo any change in 6 month follow-up. Patient had earlier suffered from recurrent OME.

In Group 1, the mean air–bone gap (ABG) improved by 3 dB (*p* = 0.023) and in Group 2 the mean ABG improved by 9 dB (*p* = 0.035). Ears with clinical improvement in Group 1 (11/25 ears, 44%) were considered to be those subjects with postoperative normalized type A tympanogram (improvement in tympanometry). In these cases the mean improvement of ABG was 7 dB (*p* = 0.011). Ears with clinical improvement in Group 2 (10/18 ears, 56%) were those subjects with only air in middle ear postoperatively, in these cases the mean improvement of ABG was 18 dB (*p* = 0.005) (Table 3).

**Table 3:** Preoperative and postoperative hearing levels in all ears and improved ears

		Hearing level (dB)		*p* value
		Preoperative	6 months postop	
All ears				
Group 1, 25 ears	PTA mean (STD)	20 (12)	17 (13)	0.115
	ABG (mean)	9	6	0.023
Group 2, 18 ears	PTA mean (STD)	40 (16)	31 (15)	0.049
	ABG (mean)	24	15	0.035
Clinically improved ears				
Group 1, 11 ears	PTA mean (STD)	17 (10)	10 (9)	0.010
	ABG (mean)	9	2	0.011
Group 2, 10 ears	PTA mean (STD)	39 (19)	21(7)	0.005
	ABG (mean)	24	6	0.005

After 6 month follow-up the mean total GBI was 14 showing the positive impact of BET in both groups, see Table 4.

**Table 4:** Quality-of-life results (GBI scores) 6 months after BET

	GBI total	General	Physical health	Social support
All ears 43 ears				
Group 1, 25 ears	13	3	12	1
Group 2, 18 ears	15	5	13	0
Clinically improved ears 21 ears				
Group 1, 11 ears	16	6	9	2
Group 2, 10 ears	19	11	13	− 4

## Discussion

In this study, we demonstrated that BET can objectively improve conductive hearing loss in patients with chronic OETD. Eleven (44%) of 25 patients with tympanic membrane retraction and ten (56%) of 18 patients MEE showed improvement in audiometric outcomes postoperatively. The mean reduction in ABG was 9 dB in patients with MEE, which is in line with the previous outcomes in the study of McMurran [3]. They showed 22 dB mean ABG preoperatively, 15 dB mean ABG postoperatively and 8 dB mean reduction in ABG. Their study population included 36 Eustachian tubes in 25 patients, of which four ears were normal in preoperative otomicroscopic examination (with only patients’ self-reported experience of chronic OETD symptoms), three ears had a grommet or T-tube in situ and all other ears had MEE with or without TM retraction. Resolution or improvement of conductive hearing loss was seen in 16 ears (62%) in their study. Our study also showed improvement in audiometric findings in patients with tympanic membrane retraction. These patients had mild conductive hearing losses with mean preoperative ABG of 9 dB and postoperative gap of 6 dB, mean reduction in ABG of 3 dB.

Our results showed that BET can improve the quality of life in OETD patients, the mean of the total GBI 14 indicates a positive influence in patients’ overall quality of life after BET as well as in subscores of general and physical health. These findings are in line with the study of Bast [7]. GBI has also shown improvement in QoL with other otorhinolaryngological interventions. GBI makes possible to compare of impact of different surgical interventios. However, the drawback is that it is not specific to OETD patients. For example, in patients with nasal polyps or sinusitis who underwent functional endoscopic sinus surgery in the study of Salhab et al. [14], the median total GBI score was 11 postoperatively. In the study of tonsillectomy for recurrent pharyngitis [15], improvements were demonstrated with the median of GBI total score of 27 and the GBI physical health score 83. Härkönen et al. [16] showed that cochlear implantation in patients with single-sided deafness had a positive influence on QoL; the mean score for total GBI was 28 at 1 year follow-up.

We demonstrated objectively that 80% of patients with TM retraction and 67% of patients with MEE were able to perform the Valsalva maneuver after tuboplasty compared to 28% in both groups preoperatively. These results in ears with TM retraction are similar to the Valsalva success rate stated in the review study of Luukkainen (80–98%) [8]. However, in our patients with MEE, the Valsalva success rate remained slightly lower compared to previous studies, but our results were confirmed objectively with an otomicroscope.

Our study showed improvement in tympanometry results in 50% of patients with TM retraction and 33% of patients with MEE. A positive trend in tympanometry results was verified already in the first postoperative visit after 1–2 weeks and it improved by the final 6 month postoperative follow-up. Our positive tympanometry results after BET are in line with previous studies (24–54% improvement in the review study of Luukkainen). In the latest meta-analysis, the average improvement of tympanometry results at the long-term follow-up was 48%, so our results are also in line with them [19]. Changes in ET inflammation scale showed that edema in ET orifice decreased after tuboplasty. Preoperatively the edema was normal to mild in 60% of cases in Group 1 and 44% in Group 2 and postoperatively it was 92% and 83%. The study's inflammation scale results align with prior findings. In the study by Silvola et al. preoperatively, 43% of cases had normal/mild ET inflammation scale and postoperatively this improved to 88% [13]. This highlights the BET potential in reducing ET inflammation. These results may indicate that tympanic membrane retraction is a milder sequala of OETD than MEE. Or it may indicate that tympanic membrane retraction is more mechanical problem and MEE is more inflammatory problem.

Some limitations in most previous studies have been that patient selection criteria have been quite heterogenous, follow-up methods have been variable and some patients have had only mild findings of OETD. Valsalva success rates were based only on patients’ subjective experience. Tympanometry and other clinical preoperative findings may have been normal and patients have had only subjective symptoms of OETD [8, 9, 18]. In our study the ability to inflate the middle ear by Valsalva maneuver was objectively observed and it can be considered more reliable than a patient’s self-reporting.

Our study stratified the patients into a group with TM retraction and a group with MEE, which makes the results more clinically relevant and introduces some consistency in selection criteria that has been lacking in many previous studies. However, it's important to note that with two subgroups, the number of patients in each of these two groups was reduced, which is a limitation of our study. Two different types of homogenous patient cohorts can help to differentiate patient characteristics that may benefit the most from BET. Our study lacked patients with baro-challenge induced ET dysfunction, which could have constituted another potential subgroup. The outcome results (Valsalva, tympanometry and ET inflammation scale) were better in patients with TM retraction compared to results of patients with MEE. But still, the limitations of this study are small number of participants with limits on statistical analysis.

It remains uncertain whether tympanocentesis may be beneficial in long-term outcomes with BET for MEE. It has been stated in a recent study of 29 ears by Formánková et al. [20] that no significant difference was reported in comparison of BET with or without tympanocentesis in patients with chronic otitis media with effusion. They also showed that the success rate of BET was lower with effusions than in other pathologies of middle ear caused by OETD. In ears with effusions, results at 6 month follow-up showed improvements in tympanometry in 48%, Valsalva success rate was 41% and audiometry in 62%. These results are in line with our outcomes in Group 2 (MEE patients) as we demonstrated 33% improvement in tympanometry, 67% success rate in Valsalva maneuver and 56% in audiometry results.

BET may be more effective in relieving negative pressure without effusion compared to presence of effusion and this should be considered with regard to postoperative expectations. It may be that MEE represents the consequences of a greater burden of obstructive disease or it may be that a primary middle ear inflammatory or allergic condition may be involved in some cases. The present results showed a trend of increased mucosal inflammation within the mucosa of the ET in MEE cases. Of note, our patients with middle ear effusion often demonstrated the best improvement of hearing outcomes. Adjunctive procedures were not used in this study and it is possible that appropriate other interventions may have improved clinical results. In a recent multicenter real-world study of 154 patients by Standring et al. [21] in which adjunctive procedures were employed in several cases, minimum clinically important improvement was reported in 85%. The present study was done in the absence of adjunctive procedures to isolate the benefit of BET alone. Short and long-term success are dependent on adequate pre- and post-operative management of predisposing underlying diseases. BET can be combined with adjunctive procedures when indicated.

## Conclusion

BET was demonstrated to be an effective treatment for chronic OETD in this study in which adjunctive procedures were not employed. Significant improvements were seen for conductive hearing loss and overall quality of life in both the tympanic membrane retraction and middle ear effusion groups. Postoperative Valsalva maneuver success rate and improvement in tympanometry were higher in tympanic membrane retraction group, but the hearing improvement was better in the middle ear effusion group. BET may be considered as a primary treatment for OETD, both in TM retraction and MEE.

## Data Availability

Anonymised data is available for systematic review if needed.

## References

[1] Shan A, Ward BK, Goman AM, Betz JF, Reed NS, Poe DS, Nieman CL (2019). Prevalence of eustachian tube dysfunction in adults in the United States. JAMA Otolaryngol Head Neck Surg.

[2] Gürtler N, Husner A, Flurin H (2015). Balloon dilatation of the Eustachian tube: early outcome analysis. Otol Neurotol.

[3] McMurran AEL, Hogg G, Gordon S, Spielman PM, Jones SE (2020). Balloon eustachian tuboplasty for eustachian tube dysfunction: report of long-term outcomes in a UK population. J Laryngol Otol.

[4] McCoul ED, Anand VK, Christos PJ (2012). Validating the clinical assessment of eustachian tube dysfunction: the Eustachian tube Dysfunction Questionnaire (OETDQ-7). Laryngoscope.

[5] Teklu M, Kulich M, Micco AG, Brindisi K, Ferski A, Niekamp K, Price CPE, Tan BK (2020). Measuring the health utility of chronic eustachian tube dysfunction. Laryngoscope.

[6] Hendry J, Chin A, Swan IRC, Akeroyd MA, Browning GG (2016). The glasgow benefit inventory: a systematic review of the use and value of an otorhinolaryngological generic patient-recorded outcome measure. Clin Otolaryngol.

[7] Bast F, Frank A, Schrom T (2013). Balloon dilatation of the eustachian tube: postoperative validation of patient satisfaction. J Otorhinolaryngol.

[8] Luukkainen V, Kivekäs I, Silvola J, Jero J, Sinkkonen ST (2018). Balloon eustachian tuboplasty: systematic review of long-term outcomes and proposed indications. J Int Adv Otol.

[9] Huisman JML, Verdam FJ, Stegman I, de Ru J (2018). Treatment of eustachian tube dysfunction with balloon dilatation: a systematic review. Laryngoscope..

[10] Poe D, Anand V, Dean M, Roberts WH, Stolovitzky JP, Hoffmann K, Nachlas NE, Light JP, Widick MH, Sugrue JP, Elliott CL, Rosenberg SI, Guillory P, Brown N, Syms CA, Hilton CW, McElveen JT, Singh A, Weiss RL, Arriaga MA, Leopold JP (2018). Balloon dilation of the eustachian tube for dilatory dysfunction: a randomized controlled trial. Laryngoscope.

[11] Meyer TA, O'Malley EM, Schlosser RJ (2018). A randomized controlled trial of balloon dilation as a treatment for persistent eustachian tube dysfunction with 1-year follow-up. Otol Neurotol.

[12] Kivekäs I, Pöyhönen L, Aarnisalo A, Rautiainen M, Poe D (2015). Eustachian tube mucosal inflammation scale validation based on digital video images. Otol Neurotol.

[13] Silvola JT, Kivekäs I, Poe D (2014). Balloon dilatation of the cartilaginous portion of the eustachian tube. Otolaryngology Head and Neck Surgery.

[14] Salhab M, Matai V, Salam MA (2004). The impact of functional endoscopic sinus surgery on health status. Rhinology.

[15] Koskenkorva T, Koivunen P, Läärä E, Alho O-P (2014). Predictive factors for quality of life after tonsillectomy among adults with recurrent pharyngitis: a prospective cohort study. Otolaryngol.

[16] Härkönen K, Kivekäs I, Rautiainen M, Kotti V, Sivonen V (2015). Single-sided deafness: the effect of cochlear implantation on quality of life, quality of hearing, and working performance. ORL.

[17] Schilder AG, Bhutta MF, Butler CC, Holy C, Levine LH, Kvaerner KJ, Norman G, Pennings RJ, Poe D, Silvola JT, Sudhoff H, Lund VJ (2015). Eustachian tube dysfunction: consensus statement on definition, types, clinical presentation and diagnosis. Clin Otolaryngol.

[18] Meyer TA, O'Malley EM, Schlosser RJ, Soler ZM, Cai J, Hoy MJ, Slater PW, Cutler JL, Simpson RJ, Clark MJ, Rizk HG, McRackan TR, D'Esposito CF, Nguyen SA (2018). A randomized controlled trial of balloon dilation as a treatment for persistent eustachian tube dysfunction with 1-year follow-up. Otol Neurotol.

[19] Froehlich MH, Le PT, Nguyen SA, McRackan TR, Rizk HG, Meyer TA (2020). Eustachian tube balloon dilation: a systematic review and meta-analysis of treatment outcomes. Otolaryngol Head Neck Surg.

[20] Formánková D, Formánek M, Školoudík L, Zeleník K, Tomášková H, Chrobok V, Komínek P (2020). Balloon eustachian tuboplasty combined with tympanocentesis is not superior to balloon eustachian tuboplasty in chronic otitis media with effusion—a randomized clinical trial. Otol Neurotol.

[21] Standring RT, O'Malley EM, Greene JB, Russell JL, McCoul ED (2021). Balloon dilatation of the eustachian tube with a seeker-based device: a registry of 169 patients. Laryngoscope Investig Otolaryngol.

